# A Contemporary View on Carnot’s Réflexions

**DOI:** 10.3390/e26121002

**Published:** 2024-11-21

**Authors:** Jan-Peter Meyn

**Affiliations:** Department Physik, Friedrich-Alexander-Universität Erlangen-Nürnberg, Staudtstraße 7, 91058 Erlangen, Germany; jan-peter.meyn@fau.de

**Keywords:** Carnot cycle, waterfall analogy, heat engine, combustion engine, diffusion, waste heat, gas turbine, efficiency

## Abstract

Entropy and energy had not yet been introduced to physics by the time Carnot wrote his seminal Réflexions. Scholars continue to discuss what he really had in mind and what misconceptions he might have had. Actually, his work can be read as a correct introduction to the physics of heat engines when the term calorique is replaced by entropy and entropy is used as the other fundamental thermal quantity besides temperature. Carnot’s concepts of falling entropy as an analogy to the waterfall, and the separation of real thermal processes into reversible and irreversible processes are adopted. Some details of Carnot’s treatise are ignored, but the principal ideas are quoted and assumed without modification. With only two thermal quantities, temperature and entropy, modern heat engines can be explained in detail. Only after the principal function of heat engines is developed is energy introduced as physical quantity in order to compare thermal engines with mechanical and electrical engines and, specifically, to calculate efficiency.

## 1. Introduction

Heat engines are essential to powering modern civilization. In recent years, engines have been replaced by electric motors, and electricity is increasingly being generated from renewable sources. The total power of all heat engines will decrease with this development, but they cannot be completely replaced by electric motors. Seasonal storage of renewable energy, as well as long-distance airplanes and ships, will continue to rely on heat engines for the foreseeable future. Profound knowledge of thermodynamics is necessary not only for physicists and engineers but also for decision makers who will have to organize the energy supply of the future.

Sadi Carnot (1796–1832) presented the first physical theory of heat engines [1] in 1824. Two centuries later, he is called the *father of thermodynamics* or alike by many authors. The Carnot cycle is a cornerstone of thermodynamics, but in most textbooks, it is used only to name the proof of the maximum energy efficiency of any heat engine. Carnot did not write about numerical values for energy efficiency, nor could he, because energy had not yet been introduced into physics. In this sense, he is famous for something he did not do. Carnot introduced the principle of the heat pump, and he proposed the combined gas and steam cycle, but he is almost never cited for these important contributions.

As an author of the early 19th century, Carnot did not use the modern terms of thermodynamics, which evolved decades later. Therefore, the meanings of his terms are ambiguous and subject to debate, as summarized, for example, in Feynman’s lectures [2]. *So, it has often be said that Carnot’s logic was wrong. But his logic was quite correct. Only Clapeyron’s simplified version, that everybody read, was incorrect*. Following Callendar [3], this paper reads Carnot’s calorique as entropy. A teaching concept [4] is developed that omits energy and comes much closer to Carnot’s ideas than the standard textbooks. Since calculations are impossible, qualitative arguments are used. Only after finding technically appropriate solutions for real heat engines based on entropy arguments are some results analyzed quantitatively based on energy.

The purpose of this article is to point out the importance of Carnot’s Réflexions today for two independent reasons. First, some core ideas are under-represented in the literature. Secondly, the adoption of his thinking for the teaching of thermodynamics is promising.

## 2. Entropy as Quantity of Heat

### 2.1. Difference to Energy

In conventional thermodynamics, heat (*Q*) is measured in units of energy. According to this definition, heat is not a physical quantity on its own but just an amount of energy. When thermodynamic processes are treated in detail, a variety of special quantities with the same unit, the joule, are often needed, such as enthalpy, exergy, thermal energy, internal energy, and so on.

Callendar [3] pointed out this problem over a century ago and provided detailed explanations of its historical development. He proposed considering entropy as the quantity of heat. Nevertheless, only advanced textbooks on statistical physics use entropy as a central quantity of thermodynamics, while introductory textbooks persist in arguing with quantities of energy. Job pointed out the correspondence of entropy with the colloquial concept of heat in his undergraduate textbook [5]. Several authors have developed the concept of using entropy as a substitute for the energy-based heat term (*Q*) [6,7,8,9,10].

Whenever heat is transferred in the conventional sense of an amount of energy (*Q*), one could also say that entropy (*S*) is transferred. But only the latter is a state variable, i.e., a system that contains a well-defined amount of entropy. Obviously, this is an advantage for teaching. Entropy can flow from a body A to a body B and is, thus, contained in B. Conventional heat (*Q*) is transferred between bodies but is not contained in a body. At this point, thermodynamics begins to seem absurd to many students, especially when the conventional concept of heat lives on partially in the physical concept of heat. *The Karlsruhe Physics Course* [11] is a proven high school textbook based on entropy as the quantity of heat. Its effectiveness has been confirmed empirically [12].

Entropy, as a quantity of heat, does not require a particle model—only the observations and conclusions described below. Certainly, this macroscopically defined quantity is upwardly compatible with microscopically defined entropy in statistical physics.

### 2.2. Latent Heat

**Figure 1:**
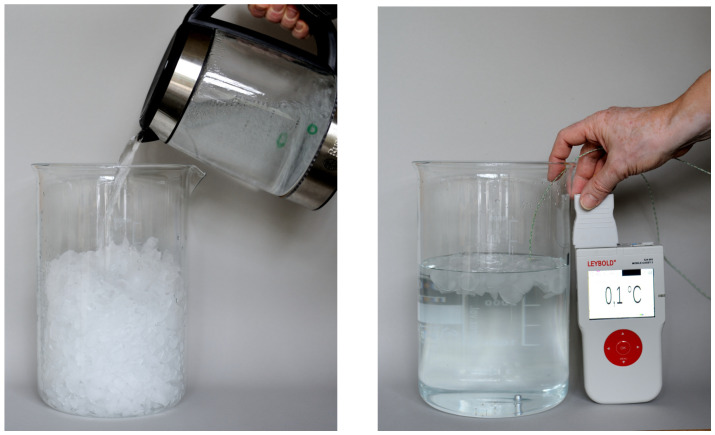
Visibly boiling water is poured into a beaker filled with crushed ice. After stirring, the mixture is ice-cold. The result cannot be explained by temperature alone because the temperature in the beaker remains constant at 0 °C.

Elementary physical quantities can usually be attributed to a phenomenon; for example, mass is a measure of inertia, pressure makes a gas more dense, electrical current has a magnetic field, and so on. Neither energy nor entropy has a specific phenomenon. In the history of science, the need for a second thermal quantity besides temperature arose relatively late. *Latent heat* was introduced by Joseph Black (1728–1799) [13] to describe the slow melting of ice. During Black’s lifetime, the nature of heat remained vague. However, Black was the first to point out the need for a quantitative measure of heat, in addition to a qualitative measure of temperature. Figure 1 shows a simple but effective demonstration experiment with a surprising result.

Most students expect a mixing temperature of the order of 50 °C because similar amounts of ice and boiling water are mixed. Usually, the teacher will find a volunteer who trusts him that the mixture will not burn his hand, but the person seems surprised to find the mixture ice cold. The explanation is based on entropy as the quantity of heat. Liquid water at 0 °C contains more entropy than solid ice at 0 °C. Boiling water at 100 °C contains more entropy than cold water. Entropy is transferred from the boiling water to the ice, which absorbs entropy as it melts.

This interpretation neglects the generation of additional entropy by mixing bodies of different temperatures. This can be accepted for now, since no student would ever come up with this problem.

### 2.3. Irreversible Process

The common statement that *heat is created by friction* can be developed into a scientific statement: Entropy is created by friction. In the scientific literature, the following synonymous formulation is commonly used: Entropy is *generated* by friction. This everyday concept can be easily experienced in the classroom when every student rubs his or her hand on the table. The scientific statement is then made by the teacher, and it is compatible with the concept of entropy as the quantity of heat. Notice that a student might say that temperature is generated by friction. But once entropy is available as the quantity of heat, it is easily accepted.

Diffusion is obviously irreversible, but does it generate entropy? A cup of hot coffee undoubtedly loses entropy to its surroundings, and this process is irreversible. But it is not obvious that additional entropy is created. This problem is solved by the following definition: *A process is irreversible if entropy is generated.* This shifts the burden of proof: the definition must be challenged by an example to the contrary, which science has never found.

In everyday life, we rely on beneficial irreversible processes. The production of goods is inevitably irreversible. For example, a pot is made from a sheet of copper. The metal is forged into the desired shape, and the new shape is permanent—hopefully! Copper is an instructive example because it can be formed at room temperature, and it becomes noticeably warmer during the forging process. This rise in temperature is a direct indication of the generation of entropy. The damping of vibrations is another class of irreversible processes that is not obvious but very important. Imagine riding a bicycle on a bumpy road without suspension.

### 2.4. Conceptual Change Towards Entropy

At the time of the publication of Carnot’s *Réflexions*, heat was often thought of as a kind of substance called *caloric*, *calorique* (French), or *caloricum* (Latin). Lavoisier (1743–1794) treated both calorique and lumière (light) as chemical reactants [14]. Empirical studies have shown that children think similarly [15].

Learning physics involves the transformation of students’ preconceptions into a scientific concept. Conceptual change is only possible if the new concept is plausible to the learner, i.e., it does not contradict fundamental beliefs [16]. Obviously, a new scientific concept can be plausible if it shares some similarities with the preconception.

**Figure 2:**
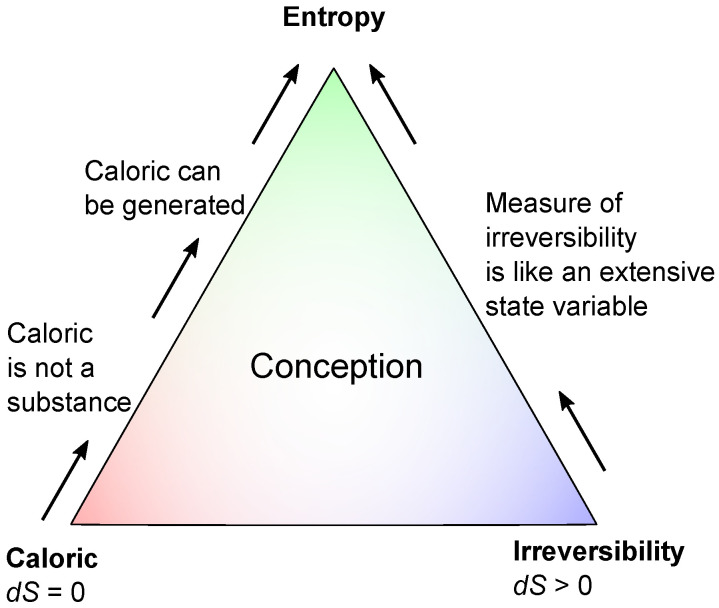
Conception of the physical quantity of entropy based on the preliminary term caloric (caloricum, calorique) and the general term irreversibility.

Entropy has a lot in common with caloric. It is contained in a body or system, it can be transferred, it can be transmitted by solids, and so on. The difference between caloric and entropy is that entropy can be generated, and this is, indeed, a difficult step. Therefore, the caloric-like aspects, on the one hand, and the generation of entropy by irreversible processes, on the other hand, should be taught separately and finally combined according to the scheme in Figure 2.

## 3. Foundation of Carnot’s Réflexions

### 3.1. Calorique as a Precursor of Entropy

Carnot could not have known what heat really is, but he was quite sure he knew, as he wrote in a footnote on page 15: *Nous jugeons inutile d’expliquer ici ce que c’est que quantité de calorique ou quantité de chaleur (car nous employons indifféremment les deux expressions), ni de décrire comment on mesure ces quantités par le calorimètre. Nous n’expliquerons pas non plus ce que c’est que chaleur latente, degré de température, chaleur spécifique, etc.: le lecteur doit être familiarisé avec ces expressions par l’étude des traités élémentaires de physique ou de chimie.* Translation [17]: *It is considered unnecessary to explain here what is quantity of caloric or quantity of heat (for we employ these two expressions indifferently), or to describe how we measure these quantities by the calorimeter. Nor will we explain what is meant by latent heat, degree of temperature, specific heat, etc. The reader should be familiarized with these terms through the study of the elementary treatises of physics or of chemistry.*

It is now widely accepted that Carnot’s text remains meaningful and correct when the term calorique is replaced by the modern term entropy [3,18,19,20]. Some scholars content that calorique is conserved, and this property is used in crucial arguments, while entropy can be generated. However, Carnot separates the flow of calorique into a reversible and a totally irreversible flow and considers only the reversible flow. Under the condition of a reversible process, the statement that entropy is conserved is correct.

**Figure 3:**
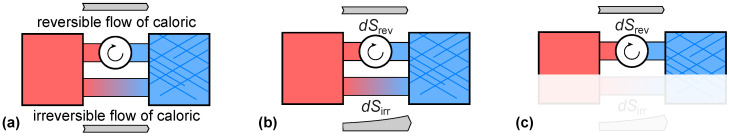
(**a**) Carnot separates the flow of calorique from a hot reservoir (red) to a cold reservoir (blue) into two fundamentally different channels: reversible flow through the reversible engine and irreversible flow through direct contact. (**b**) Replacing calorique with entropy requires a change in the irreversible flow, and entropy is generated. (**c**) For the sake of simplicity, only the upper part of (**b**) is often shown in textbooks. This obscures the intelligent separation of reversible from irreversible entropy flow.

Carnot excluded irreversible processes in order to get to the heart of heat engine physics. This is a brilliant move on the same level as the elimination of friction in mechanics. The idea is sketched in Figure 3.

Since the separation into reversible and irreversible processes works for both entropy and a preliminary term such as calorique, new students can understand the separation even without having developed a complete scientific understanding of entropy yet. The flow of calorique or entropy in direct contact should be avoided at all costs, as it does not contribute to the function of the engine.

Planck coined the term *natural* process [21] for an irreversible process, but it never became part of the scientific language. This is unfortunate because irreversible is often considered to be irregular or at least unnecessary or undesirable. Often, only the reversible process shown in Figure 3c is discussed in textbooks. Without a natural process, a heat engine seems unnatural, and some students even make fun of the “esoteric” Carnot engine.

Critics argue that Carnot treated calorique as a substance that cannot be generated [22,23,24]. The argument is valid but irrelevant to his treatment of the reversible heat engine. Entropy is not generated in a reversible process and, therefore, has the same property as calorique in this very context. This is an advantage for teaching based on Carnot’s waterfall analogy discussed in the following section, since the model also works with a preliminary conception of heat.

### 3.2. Waterfall Analogy

**Figure 4:**
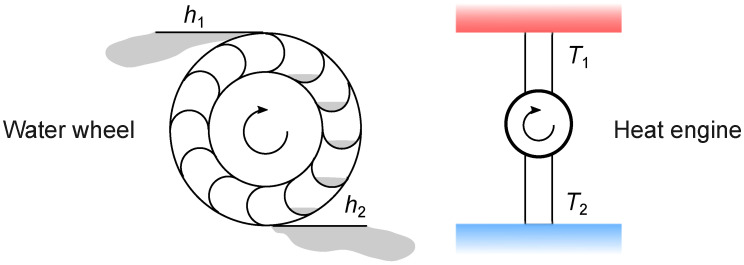
Waterfall analogy. The shaft of a water mill is driven by the mass of water falling from a height of $h_{1}$ to a lower height ($h_{2}$). In the same way, the fall of entropy from temperature $T_{1}$ to a lower temperature ($T_{2}$) drives the shaft of a heat engine.

Carnot describes the analogy between a waterfall and heat flowing through an engine (p. 28): *D’après les notions établies jusqu’à présent, on peut comparer avec assez de justesse la puissance motrice de la chaleur à celle d’une chute d’eau: toutes deux ont un maximum que l’on ne peut pas dépasser, quelle que soit d’une part la machine employée à recevoir l’action de l’eau, et quelle que soit de l’autre la substance employée à recevoir l’action de la chaleur. La puissance motrice d’une chute d’eau dépend de sa hauteur et de la quantité du liquide; la puissance motrice de la chaleur dépend aussi de la quantité de calorique employé, et de ce qu’on pourrait nommer, de ce que nous appellerons en effet la hauteur de sa chute (I), c’est-à-dire de la différence de température des corps entre lesquels se fait l’échange du calorique.* Translation: *According to established principles at the present time, we can compare with sufficient accuracy the motive power of heat to that waterfall. Each has a maximum that we cannot exceed, whatever may be, on the one hand, the machine which is acted upon by the water, and whatever, on the other hand, the substance acted upon by the heat. The motive power of the waterfall depends on its height and on the quantity of the liquid; the motive power of heat depends also on the quantity of caloric used, and on what may be termed, on what in fact we will call, the height of its fall, that is to say, the difference of temperature of the bodies between which the exchange of caloric is made.* This analogy is sketched in Figure 4. Clearly, altitude and temperature are analogous. For extensive quantities, we need to interpret the text. The amount of liquid could be measured by volume, mass, or the amount of matter. The best choice is mass in kg because it makes later energy calculations easy. The caloric quantity is entropy. Note that entropy is analogous to mass, not to water. Even in the original text, caloric is not considered a liquid material but a physical quantity. The waterfall analogy points out that the temperature difference is crucial to the performance of a heat engine, not the pressure difference, which seems more obvious for a mechanical device.

Today, entropy takes the place of caloric. Entropy generation by irreversible processes would spoil the analogy because water (or the mass of water) is clearly conserved. Therefore, it is essential to emphasize the validity of the analogy for the reversible part of the total entropy flow through a real heat engine. At first sight, this seems to be a subtle trick. However, in mechanics, we always take reversible motion for granted. Even at the university level, only a small minority of problems in a mechanics class involve friction at all, and if they do, the special effect of friction is pointed out.

The advantage of the waterfall analogy is that it emphasizes heat quantity over temperature. Water is the main thing in a waterfall, and height is a characterization. Similarly, the amount of heat, or entropy, is the main thing in a heat engine. Many laypeople assume that the amount of waste heat in heat engines can be reduced or even eliminated by clever engineering. The waterfall analogy helps to overcome this misunderstanding. The entropy that goes into the engine must come out at the same rate, at least in the case of a reversible engine. Finding a process that cools the medium to near-ambient temperature does not solve the problem of entropy emission. Steam power plants have to have huge cooling towers (unless they are on wide rivers), even though the outlet temperature of the steam turbine is of the order of 35 °C, which is not even hot in the usual sense. Carnot (p. 9f.): *L’eau froide du condenseur s’empare donc en dernier résultat du calorique développé par la combustion. Elle s’échauffe par l’intermédiaire de la vapeur, comme si elle eût été placée directement sur le foyer.* Translation: *Then, as a final result, the cold water of the condenser takes possession of the caloric developed by the combustion. It is heated by the intervention of the steam as if it had been placed directly over the furnace.*

**Figure 5:**
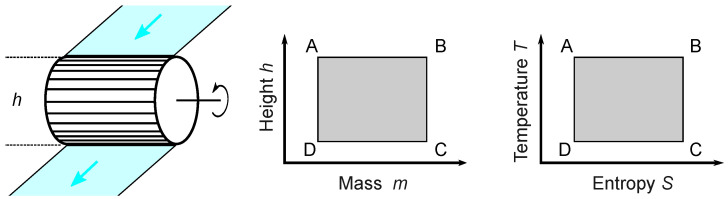
Idealized water mill process for a wheel height of *h*. The bucket is filled at maximum height from state A to state B; then, the water descends (B–C) and pours out of the bucket at a minimum height (C–D). The empty bucket is raised again (D–A). For Carnot’s idealized heat engine, there is isothermal heating from A to B, adiabatic expansion (B–C), isothermal cooling (C–D), and adiabatic compression (D–A). The motive power of a reversible water mill is proportional to the mass flow and the height difference. Therefore, the area in the h,m diagram is proportional to the motive power. Similarly, the area in the T,S diagram is proportional to the motive power of a reversible heat engine.

The mechanical output of a heat engine is proportional to the height and the current of the entropy fall. as shown in Figure 5. Carnot was not sure about the height proportionality, but this does not matter for a contemporary interpretation, where the proportionality is granted by the definition of the thermodynamic temperature [25].

If the temperature difference between the hot and cold contact of the motor is doubled, the mechanical output (Carnot: *puissance motrice*; translated as *motive power*) is doubled. Note that *motive power* has not been strictly defined in terms of energy yet.

### 3.3. Statement on Efficiency Without Energy

Looking up Carnot in the index of a typical textbook leads to a section on the maximum energy efficiency of a heat engine. This could not have been the main point in the original text simply because energy was not invented for physics until two decades later. For Carnot, the most efficient engine was easy to characterize, i.e., an engine in which the medium undergoes a reversible change of state and all irreversible processes are excluded.

**Figure 6:**
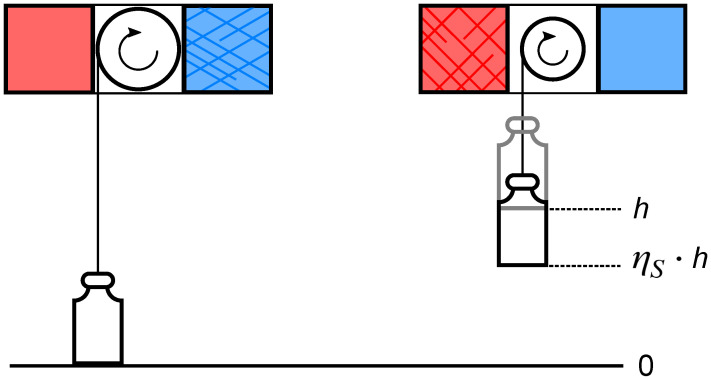
Definition of isentropic efficiency ($\eta_{S}$). The heat reservoirs contain a mixture of liquid and solids, for example, tin (505 K, red) and water (273 K, blue). The amount of entropy entering the heat engine can be determined by the mass of the solidified substance in the hot reservoir. Note that Carnot did not demand infinite reservoirs—only constant temperatures. Our suggestion of finite reservoirs based on phase mixtures is a valid development of Carnot’s concept.

Figure 6 shows the principle of characterizing a heat engine by isentropic efficiency. The engine is in contact with two reservoirs, and a given amount of entropy is transferred from the hot reservoir to the cold reservoir. The reversible engine lifts a mass (*m*) to a height of *h*. Under the same conditions, a real engine lifts the mass to a smaller value ($\eta_{S}h$) with the isentropic efficiency ($\eta_{S}$).

This looks like a quantitative definition of isentropic efficiency ($\eta_{S}$), but the height (*h*) cannot be determined experimentally because a reversible engine is only an idealization. Therefore, the modern scientist or engineer demands a definition based on energy. This obscures the simplicity of Carnot’s division into reversible and irreversible processes. Historically, this is understandable. From the beginning, heat engines were built to make money. For engineers, it was never enough to maximize efficiency; they had to predict the actual amount of fuel needed to perform a mechanical task.

Only recently, on the scale of two hundred years, has there been a shift in advanced engineering from maximizing energy efficiency to minimizing entropy generation. According to Bejan [26], the idea of entropy production minimization emerged in the 1970s and blossomed in the 1990s. He provide a wide range of examples where entropy generation minimization yields the desired results more easily than energy calculations. For entropy generation, especially in turbomachinery, see reference [27].

## 4. Combustion Engines

Based Carnot’s concept but using entropy instead of caloric we look at the optimization of modern heat engines, which is basically the reduction of unnecessary irreversible processes. Entropy is generated by

1. Flame reaction;
2. Thermal diffusion of hot flame gases;
3. Thermal diffusion of hot exhaust gasses;
4. Heat conduction at the inlet;
5. Heat conduction at the outlet;
6. Friction due to the viscosity of the medium;
7. The turbulence of the medium;
8. Leakage of the medium, mainly in turbines;
9. Mechanical friction;
10. Turbulence of the exhaust gas;
11. General operation of the engine.

The flame reaction (1) is the unavoidable generation of entropy to run the engine. Heat conduction at the boundary of the engine (4 and 5) was considered in an educational paper by Curzon and Ahlborn [28]. We return to this subject in Section 7.3, but for now, this contribution is considered small compared to others. Items 6 through 11 are engineering issues that are also neglected in a first approach. The remaining irreversible processes are the thermal diffusion of the hot flame gas (2) and the thermal diffusion of the exhaust gas (3). The latter corresponds to the common term waste heat. The hot gas emitted by an internal combustion engine cools down by diffusion in the environment without any mechanical benefit. The thermal diffusion of the hot flame gas before it becomes effective inside the machine is seldom mentioned. Contributions 1 and 2 are larger than the other items, and they predominantly determine the efficiency of a heat engine.

Carnot was clever enough to avoid the thermal diffusion of flame gases by inventing thermal reservoirs. This does not work for any real engine running on fuel. Entropy flow is closely related to material flow.

### 4.1. Stirling Engine

**Figure 7:**
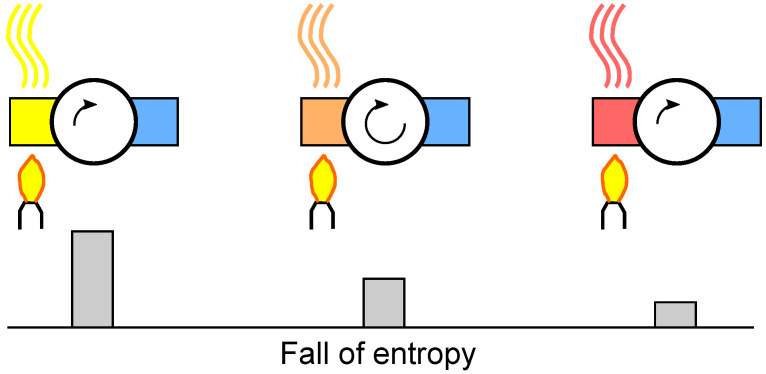
The diffusion of hot flame gases after touching the inlet of a heat engine, such as a Stirling engine, is an obvious loss. The fall of entropy is reduced when the flame gases are cooled due to more efficient entropy transfer at the hot end of the heat engine.

According to Carnot’s waterfall analogy, the hot inlet of the Stirling engine should be at the highest possible temperature, and the cold end should be at the lowest possible temperature, which is the ambient temperature. The latter is easily achieved with cooling water. But the hot end poses a problem: After being in contact with the engine, the temperature of the flame gas is at least as high as the temperature of the engine’s hot end. Most of the flame-gas entropy does not enter the engine but is dissipated into the environment. If the inlet temperature is lower, a larger entropy current enters the engine. However, the fall of entropy is less in this case (see Figure 7). If the flame gas is perfectly cooled, the entropy fall is infinitely small. The optimum temperature of the engine inlet is the geometric mean of the flame temperature and the outlet temperature. The proof [29,30] is based on energy.

### 4.2. Gas Turbine as Prototype of a Combustion Engine

**Figure 8:**
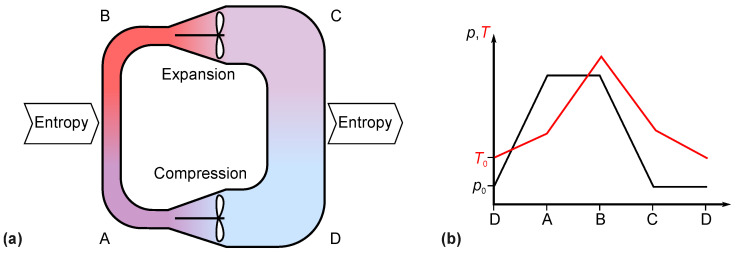
Gas turbine cycle. (**a**) Flow in a hypothetical closed-cycle gas turbine. The compressor is driven by the expander turbine on the same shaft in a real turbine. (**b**) Temperature and pressure as a function of the path through the closed tube.

Combustion engines use internal entropy production by burning fuel inside a combustion chamber. The torque is transmitted to the outside, either by a crankshaft driven by pistons or by a turbine rotor. We will not discuss the subtle differences in the thermodynamic cycles of diesel and gasoline piston engines but treat the gas turbine as a prototype. Demanding, high-power applications such as power plants and aviation always favor turbines over reciprocating engines. A hypothetical closed-cycle gas turbine such as that shown in Figure 8 would have the same hot gas diffusion problem as a flame-heated Stirling engine—at least at first glance.

**Figure 9:**
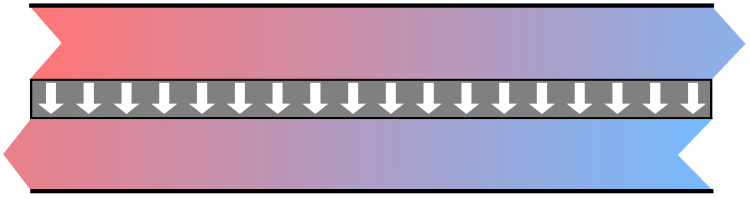
Countercurrent heat exchange. The upper fluid stream on the right and the lower fluid stream on the left cannot mix but are in contact with thin metal sheet (gray). At each point of the sheet, the temperature is slightly higher in the upper stream than in the lower stream. Therefore, entropy flows from top to bottom (white arrows).

In contrast to a Stirling engine, a steady stream of gas flows through the gas turbine. This allows for complete entropy transfer through a countercurrent heat exchanger at both the entropy input and entropy output of the closed-cycle gas turbine. The temperature of the flame gas is cooled to ambient temperature at the limit of the reversible process. The principle of countercurrent heat exchange is shown in Figure 9.

A real gas turbine is open between points C and D, as shown in Figure 8, and the fuel is burned internally. This sets the pressure in front of the compressor and behind the expander to ambient pressure, but otherwise, the construction has two major advantages. First, heat resistance of the walls is absent because there are no walls between the flame and the medium. Secondly, the construction is simple and lightweight. This is why gas turbines are used in aviation. Diesel and gasoline engines share the first advantage of internal combustion in spite of their oscillating internal motion and some differences in the course of pressure and temperature.

The exhaust gas of an internal combustion engine has a high temperature of several hundred °C, and diffusion of the hot exhaust gas is a severe, irreversible process. However, the situation is better than for a Stirling engine, since the flame-gas temperature is significantly lowered by adiabatic expansion. Adiabatic expansion is an isentropic or reversible process. The exhaust-gas temperature is higher than expected from simple calculation because the adiabatic coefficient is lower than for fresh air due to the high CO_2_ and H_2_O contents.

### 4.3. Steam Turbine

A lower outlet temperature than from an open gas turbine can only be achieved at lower-than-ambient pressure. This requires a closed cycle of the medium, which introduces two new problems. First, an entropy current resistance is introduced, and secondly, the entropy density is low due to the low pressure. The situation becomes much more favorable when the medium undergoes a phase transition near ambient temperature, as in the steam cycle, which is also called the Clausius–Rankine cycle. The entropy at the outlet of the engine is removed from the liquid phase rather than the gaseous phase, so the entropy density is much greater.

**Figure 10:**
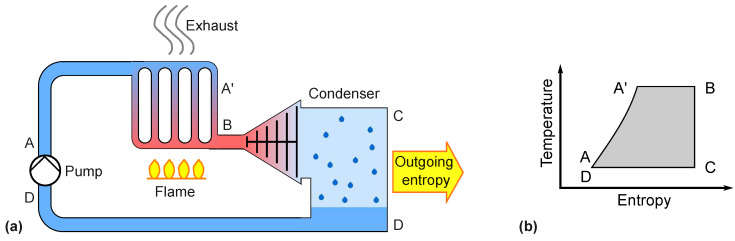
(**a**) Water and steam flow in an idealized steam turbine. (**b**) Corresponding Clausius–Rankine cycle in the T,S diagram. At point D, water is at low pressure and low temperature. The feed pump raises the pressure with a barely noticeable increase in temperature at point A. The water temperature increases by absorption of entropy in the countercurrent heat exchanger until the boiling temperature is reached at A’. Further entropy is absorbed during evaporation until the fully gaseous state is reached at B. Adiabatic expansion lowers the temperature to near ambient temperature (C) without changing the entropy. Entropy is discharged to a river or cooling tower at ambient temperature until the cycle is completed in D.

**Figure 11:**
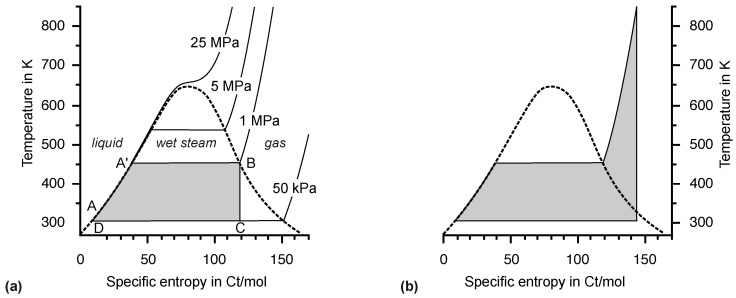
Clausius–Rankine cycle in the T,s diagram at 1 MPa pressure. (**a**) A cycle based on steam at the boiling temperature does not work because after adiabatic expansion to point C, the the water-droplet content in the wet steam is much too high. (**b**) After heating the steam the maximum temperature of 850 K, the steam contains sufficient entropy to reach the phase boundary after adiabatic expansion to 310 K. The unit of entropy is named Carnot (Ct) [3,11]. Energy is not introduced yet; therefore, the common unit of J/K is not appropriate, and kgm^2^s^−2^K^−1^ seems too complicated for a basic quantity. As in the technical literature, the specific entropy (*s*) in Ct/mol is used on the axis of abscissas.

**Figure 12:**
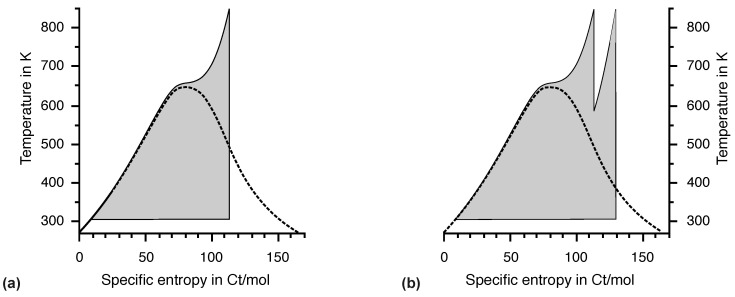
Clausius–Rankine cycle at 25 MPa pressure. (**a**) Compared to the situation in Figure 11b, the maximum temperature is reached at lower specific entropy. Therefore, the same problem of excessive liquid water content in the cold steam arises. (**b**) Intermediate expansion to the vicinity of the phase boundary and subsequent heating to the maximum temperature, again, shift the vertical line to a higher entropy value. During adiabatic expansion, the phase line is crossed. Whether such a small proportion of liquid water is tolerable or a second intermediate heating becomes necessary is decided by real steam turbine experts.

The temperature at the outlet of a steam turbine is on the order of 308 K = 35 °C, depending on the climate at the power plant site. This is close enough to ambient temperature for our purposes. The entropy of the gaseous water enters the condenser at a constant temperature. This is represented by the lower horizontal line in the T,s diagrams (Figure 10, Figure 11 and Figure 12). In terms of entropy emission at ambient temperature, real steam turbines are close to the theoretical optimum.

The advantage of the phase transition at the low temperature of the steam cycle entails a disadvantage at high temperatures. Water receives considerable entropy at a constant boiling temperature, which corresponds to a wide but not very high entropy fall in the T,s diagram presented in Figure 11a.

Figure 11b illustrates the need for further heating of the steam after complete vaporization. The adiabatic expansion in the turbine, represented by the vertical line, must start at a high entropy value in order to end near the phase boundary. Otherwise, as in the preliminary cycle presented in Figure 11a, there would be too much liquid water in the turbine, which would damage the turbine blades. Further heating after evaporation also adds a small portion of high-entropy fall, but this is not the compelling reason for it.

A higher pressure provided by the feed pump results in a higher boiling temperature and, hence, higher entropy fall. Technical problems associated with high pressure have been a problem associated with steam engines from the beginning. A substance with a higher boiling temperature facilitates entropy supply makes a phase transition at ambient temperature impossible. The low pressure of water in the condenser of 50 kPa is already difficult to handle. Its thermodynamic properties make water the best choice for the steam cycle, not its low price. The maximum temperature of 850 K and maximum pressure of 25 MPa are limited by available materials. Only minor improvements are expected in the future. Figure 12a shows the effect of increasing the pressure to 25 MPa in the T,s diagram. The maximum temperature is reached at a lower specific entropy. Again, adiabatic expansion would result in a large fraction of liquid water in the turbine, so additional heating is required. Since the maximum temperature has already been reached, the steam must be cooled by adiabatic expansion near the dew line and reheated to the maximum temperature. Note that these technical details can be understood without arguments based on energy but based on temperature and entropy, as well as the concept of the phase diagram. Obviously, the cross section of the entropy fall is much larger at high pressure, and so is the driving power of the steam turbine. However, the highest possible temperature of 850 K is still far from optimal.

The flame temperature of carbon- or hydrogen-based fuels is on the order of 2000 K, and the effective input temperature of a gas turbine is 1640 K. The latter value is the benchmark that a steam turbine should achieve but cannot because of material limitations.

### 4.4. Combined Cycle Turbine

Carnot invented the combined cycle engine (p. 109f.): *5°. Un des inconvéniens les plus graves de la vapeur est de ne pouvoir pas être prise à de hautes températures sans nécessiter l’emploi de vaisseaux d’une force extraordinaire. Il n’en est pas de même de l’air, pour lequel il n’existe pas de rapport nécessaire entre la force élastique et la température. L’air semblerait donc plus propre que la vapeur à réaliser la puissance motrice des chutes du calorique dans les degrés élevés; peut-être dans les degrés inférieurs la vapeur d’eau est-elle plus convenable. On concevrait même la possibilité de faire agir la même chaleur successivement sur l’air et sur la vapeur d’eau. Il suffirait de laisser à l’air, après son emploi, une température élevée, et, au lieu de le rejeter immédiatement dans l’atmosphère, de lui faire envelopper une chaudière à vapeur, comme s’il sortait immédiatement d’un foyer.* Translation: *(5) One of the gravest inconveniences of steam is that it cannot be used at high temperatures without necessitating the use of vessels of extraordinary strength. It is not so with air for which there exists no necessary relation between the elastic force and the temperature. Air, then, would seem more suitable than steam to realize the motive power of falls of caloric from high temperatures. Perhaps in low temperatures steam may be more convenient. We might conceive even the possibility of making the same heat act successively upon air and vapor of water. It would be only necessary that the air should have, after its use, an elevated temperature, and instead of throwing it out immediately into the atmosphere, to make it envelop a steam-boiler, as if is issued directly from a furnace.*

This is one of the most remarkable statements in Carnot’s Réflexions because he predicted a solution that became a mature technology only thirty years ago. The combined cycle proposal is not even mentioned in articles devoted to Carnot’s personal achievements [22,31,32].

**Figure 13:**
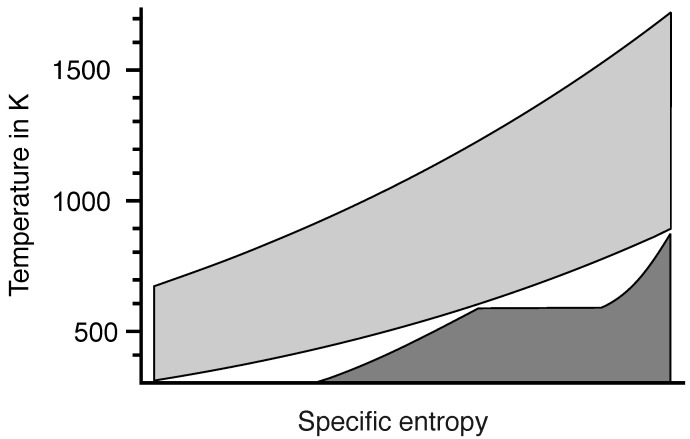
T,s diagram for the gas turbine (light gray) and steam turbine (dark gray) of a combined cycle engine. Minimizing the gap between the two areas in the figure is an obvious requirement.

The combination of a gas turbine and steam turbine on a common shaft with an electric generator is the principal construction for modern power plants, including future hydrogen power plants. Figure 13 showed the T,s diagram for a typical combined cycle engine.

## 5. Heat Pump

Carnot invented the heat pump (p. 16): *Réciproquement partout où l’on peut consommer de cette puissance, il est possible de faire naître une différence de température, il est possible d’occasioner une rupture d’équilibre dans le calorique.* Translation: *Reciprocally, wherever we can consume this power, it is possible to produce a difference of temperature, it is possible to occasion destruction of equilibrium in the caloric.*

Thomson wrote about heat pumps in 1854 without citing Carnot [33]. But earlier, he recognized the function of a reversible heat engine as heat pump in his seminal paper on the temperature scale [25]; a review of Carnot’s work [34]; and a series of review papers on the dynamical theory of heat, starting with ref. [35]. Presumably, Thomson did not think about using a real heat pump before writing his 1854 paper, but he eventually investigated both cooling and heating.

Artificial refrigeration was developed in the 19th century, but it seems to have been a practical issue from the beginning, so the cold inlet of a refrigerator received much more attention than the hot outlet. The first refrigerators were based on an inverse steam cycle, as are modern household refrigerators. Stirling engines in heat-pump mode have been used for experimental cooling since 1941 and for commercial air liquefaction since 1955 [36]. Apparently, Carnot’s contribution had been forgotten by then.

Heat pumps for room heating emerged in the 1970s. Today, the majority of newly built single-family homes in Germany are equipped with a heat pump [37]. At first glance, using a heat engine to drive a heat pump instead of burning fuel directly, as humans have done for thousands of years, seems complicated. However, both the heat engine and the heat pump are ideally reversible; in practice, the reversible part of the entropy flow is significantly larger than the irreversible part. In a conventional furnace, entropy is generated at high temperatures, as in a heat engine, but the final temperature is reached by irreversible diffusion.

**Figure 14:**
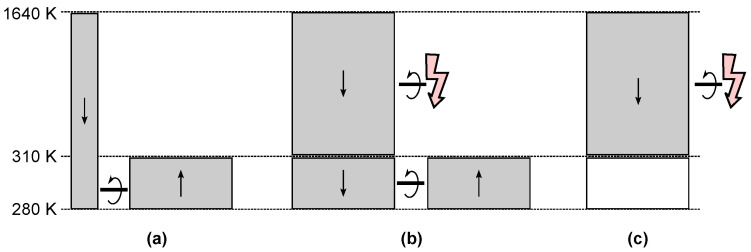
Fall of entropy of a reversible heat engine (arrow down) and heat pump (arrow up). The temperature values of 310 K (37 °C) and 280 K (7 °C) are theoretical values for room heating and ambient temperature, respectively, not technical values. (**a**) A heat pump is driven by a reversible heat engine. (**b**) The current of the entropy fall, represented by the width of the rectangle, is increased to the current of the reverse entropy fall of the heat pump. The reversible heat engine supplies additional electricity to the power distribution network. The entropy flow of the engine is divided into an upper part and a lower part. We imagine a high-temperature turbine and a low-temperature turbine for the temperature interval pf 310 K to 280 K, but this is not mandatory for the argument. The low-temperature part of the entropy flow of the engine is used to pump the entropy back to the temperature of 310 K. (**c**) Since all machines are reversible, the low-temperature engine/pump combination can be removed. Instead, an entropy flow of the same magnitude can be fed directly into the space heating system. This is the principle of cogeneration.

The use of combined heat and power, also called cogeneration, is an alternative method of space heating. One may naively assume that the temperature at the outlet of the power plant would be high enough to keep a nearby living room comfortably warm. In this approximation, the heat would be free. In practice, however, a larger district heating network is required to absorb the large amount of entropy from an industrial power plant. The inlet temperature of the heating network should be on the order of 90 °C. Therefore, the fall of entropy of the steam turbine ends at a temperature of 90 °C instead of 35 °C. The mechanical or electrical output per unit of fuel becomes smaller. Theoretically, cogeneration is as efficient as producing electricity to drive a heat pump, since in both cases, the diffusion of entropy is limited to an unavoidable amount (see Figure 14). The choice of either heating scheme depends on practical arguments. Cogeneration is more suitable for high-density housing, while heat pumps are preferred for single-family homes. Theoretically, both heat pumps and cogeneration are perfect.

## 6. Energy

### 6.1. Quantitative Comparison

So far, we have covered the basics and some interesting details of heat engines based on temperature and entropy. Energy was not necessary to obtain the results. Historically, energy was invented to compare mechanical and thermal processes. Joule determined the temperature rise of an amount of water falling down 772 feet in the Earth’s gravitational field to be 1 °F [38]. In metric values, a volume of water falling irreversibly from a height of 423 m heats up by 1 K. The experimental achievement was an irreversible slowing of the falling mass by stirring the water in the famous paddle wheel barrel, while the rest of the apparatus ran almost without friction.

The potential energy decreases at a rate of dE/dt, and simultaneously, entropy is generated at a rate of dS/dt. The decrease in potential energy is equal to the energy of the generated entropy, usually in the following differential form :

(1) dE=TdS.

### 6.2. Energy Conservation

**Figure 15:**
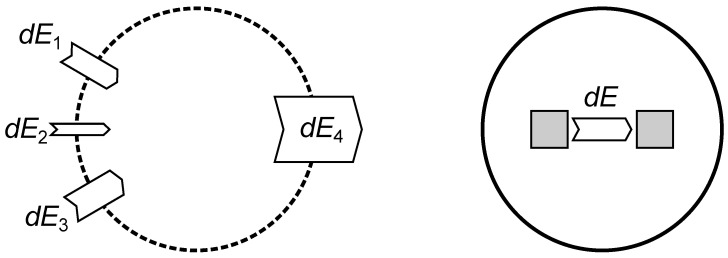
Different energy influxes ($dE_{1}$, $dE_{2}$, and $dE_{3}$) into an open system (dashed line) are balanced by an energy outflux ($dE_{4}$). The amount of energy within the open system is constant if the sum of energy fluxes is zero: $\sum_{i}dE_{i}=0$. Many elementary physics books formulate a special case in which energy is constant in a closed system, as shown on the right. This case is more convenient for physicists who have the freedom to define a sufficiently large enclosure but less useful for engineers, who have to deal with open systems in reality.

Reversible processes were quantitatively compared long before the energy conservation law was found [24]. Only with the inclusion of irreversible processes by the mechanical heat equivalent was the comparison of mechanical, electrical, and thermal processes complete. Chemical processes were added later by Gibbs, but they were included implicitly as soon as the law of conservation of energy was accepted as a fundamental physical law. Figure 15 illustrates the conservation of energy in open and closed systems.

The strength of the conservation of energy law, namely the quantitative evaluation of reversible and irreversible processes alike, is also its weakness with respect to the common notion of energy consumption. The conservation law does not distinguish between first and second quality energy.

In contrast, the mechanical heat equivalent is often called a clarification of what heat really should be, namely an amount of energy. This is unfortunate because it makes it very difficult to explain the everyday concept of *energy consumption*. Therefore, introductory physics textbooks usually do not clarify the meaning of energy consumption through a definition based on scientific terms. This bold statement can be confirmed by consulting the index or searching the digital text. Engineering books cannot tolerate this deficiency. A common way out is to use the technical terms exergy and anergy [39].

### 6.3. Energy Consumption or Degradation

Carnot did not need to talk about energy consumption because energy had not yet been invented. Section 4 illustrates some important heat engine physics without energy. However, any contemporary introduction to thermodynamics must include energy as one of the most fundamental concepts of physics and, of course, for quantitative analysis. Therefore, we propose the following definition. *Energy is consumed by irreversible processes.* According to Equation (1), the rate of energy consumption ($dE_{irr}$) is given by the rate of produced entropy ($dS_{irr}$) and the thermodynamic temperature (*T*).

(2) $$dE_{irr}=TdS_{irr}$$

This equation is also known as the Gouy–Stodola law [40,41]. Energy consumption and energy degradation [42] are synonyms.

### 6.4. Energy Supply

Having defined the physical meaning of the everyday term energy consumption, we need to clarify energy supply. Finding good words is primarily a linguistic problem, but science should respect everyday language as a basis for communication. Successful learning of physics is only possible if the new scientific concept is understandable from the student’s point of view [16].

The terms production and generation are inappropriate because these words are synonyms for creation. For example, entropy is generated by irreversible processes. The source is ambiguous. The water gushing out of a natural spring existed before, but the light emitted from a light source is created. Although energy source is a common term, energy supply seems more appropriate for teaching. These language problems are different in German, the native language of the author. Accordingly, the use of scientific terms in any other language must be viewed critically.

## 7. Energy Analysis of Heat Engines

The aim of this paper is to show that Carnot’s Réflexions provide much more than the principle of the ideal heat engine. They contain the idea of how to build optimal real heat engines under the given technical boundary conditions: irreversible processes such as thermal diffusion, mixing of cold and hot substances, mixing of fast and slow moving substances, etc., must be reduced as much as possible. Today, we are not satisfied with finding the best practical heat engine, but we insist on a measurable quantitative indication of efficiency based on energy.

### 7.1. Physical Origin of Non-Unity Efficiency

The ratio of the mechanical or electrical output power ($dE_{use}$) of a heat engine to the total power ($dE_{1}$) associated with the entropy current (dS) at the inlet of the engine is commonly referred to as the Carnot efficiency ($\eta_{C}$). Since no entropy is generated in the reversible engine, the entropy current (dS) remains constant throughout the process. The power of the entropy current at the output is $dE_{2}$. The energy efficiency is expressed as

(3) $$\eta_{C}=dE_{use}/dE_{1}=\left(dE_{1}-dE_{2}\right)/dE_{1}=\left(T_{1}dS-T_{2}dS\right)/T_{1}dS=1-\frac{T_{2}}{T_{1}}.$$

Why is the efficiency ($\eta_{C}$) less than one, even though the Carnot cycle is reversible? Different answers have been given to this question [43]. The problem stems from the assumption of infinite thermal reservoirs at the inlet and outlet of the Carnot engine. The high-temperature reservoir contains infinite entropy, and the flow rate through the machine approaches zero. Under these conditions, it seems safe to say that the temperature of the high-temperature reservoir would never change; entropy seems to be there at no cost.

Less philosophical and easier for beginners to understand is the assumption of finite reservoirs. Entropy flowing through the heat engine must eventually be replaced in the hot reservoir. New entropy must be created by an irreversible process. Therefore, some energy is consumed before the associated entropy enters the engine. The general guideline for building a heat engine is to have your cake and eat it, too. Without entropy, the engine will not work, but otherwise, as little entropy as possible should be generated in order to achieve high energy efficiency.

Real internal combustion engines have no entropy reservoir at the inlet. This does not spoil the argument because it is independent of a reservoir. We like to think that heat enters the heat engine, but in fact, cold fuel enters the heat engine, which irreversibly reacts to hot gas inside. For gas turbines, this is obvious, but for steam turbines or Stirling engines, the open system boundary has to be chosen accordingly. If the enthalpy of methane is used in a calculation, the thermodynamic process starts with cold methane and air but not with a hot mixture of CO_2_ and H_2_O.

### 7.2. Utilization of Entropy Generated by Diffusion

The temperature difference of the entropy fall in a combined cycle power plant of 1330 K (1640 K–310 K) is more than twice as large as the temperature difference in a steam power plant with a temperature difference of 560 K (870 K–310 K), but for a given fuel input rate, the output power ratio is only 1.3. For perfect reversible engines in contact with heat reservoirs, the problem is very similar with $\eta_{C}=0.82$ for an entropy fall from 1640 K to 310 K and $\eta_{C}=0.65$ for an entropy fall from 870 K to 310 K. In the following, a reversible Carnot engine is assumed for simplicity.

Let $T_{0}=1640$ K be the highest possible temperature for the given fuel. Any lower inlet temperature must be a result of irreversible diffusion due to thermal resistance or mixing of hot and cold gases. In this case, the engine is driven not only by the initial entropy of the fuel but also by the entropy generated by all possible irreversible processes that take place before the entropy reaches the working fluid.

**Figure 16:**
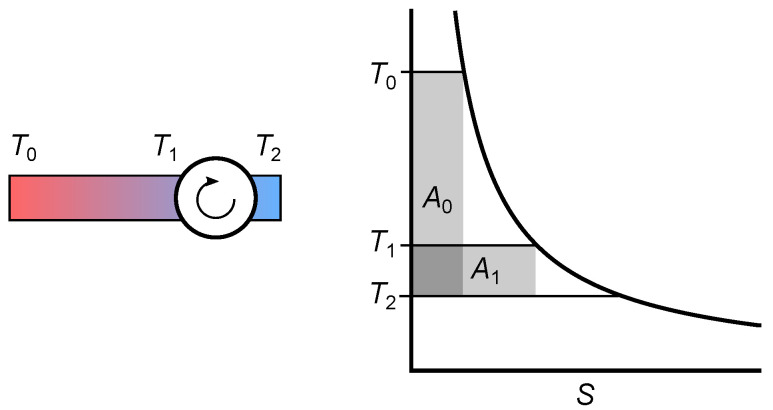
A thermal resistor is assumed as a cause of irreversible temperature reduction. The entropy produced in the thermal resistor increases the entropy current into the engine, ass represented by an increased width of area $A_{1}$.

Areas $A_{0}$ and $A_{1}$ in the T,S diagram presented in Figure 16 represent the output power of the engines at inlet temperatures $T_{0}$ and $T_{1}$, respectively. The outlet temperature ($T_{2}$) is the same for both engines. The ratio of $A_{0}$ to $A_{1}$ is

(4) $$\frac{A_{1}}{A_{0}}=\frac{dS_{1}\left(T_{1}-T_{2}\right)}{dS_{0}\left(T_{0}-T_{2}\right)}=\frac{T_{0}\left(T_{1}-T_{2}\right)}{T_{1}\left(T_{0}-T_{2}\right)}=\frac{1-\frac{T_{2}}{T_{1}}}{1-\frac{T_{2}}{T_{0}}}<1.$$

Even though the entropy from the irreversible diffusion before the inlet of the engine is used, the output power is always less than for an engine with the highest possible inlet temperature. Obviously, energy analysis provides a more accurate result than estimating the heights of the entropy falls. This is one of many examples where energy is indispensable for engineers.

This result does not make the waterfall model a bad choice. A beginner who knows only about temperature and entropy can understand the technical details of modern heat engines, as shown in Section 4. On the other hand, someone who knows temperature and energy but not entropy would have a hard time defining the meaning of the terms energy consumption or degradation or loss.

### 7.3. Endoreversible Engines

In a seminal paper [28], Curzon and Ahlborn showed that the efficiency of a heat engine in which the working medium undergoes reversible and irreversible state transition caused by heat conduction at the interface between the thermal reservoirs and the working medium at maximum output power can be expressed as follows:

(5) $$\eta_{CA}=1-\sqrt{\frac{T_{2}}{T_{1}}}$$

Equation (5) has been found several times, then forgotten [44,45]. For a small $T_{2}/T_{1}$, the efficiency ($\eta_{CA}$) is half of Carnot’s efficiency. Heat engines that cycle in finite time with a reversible state change of the working medium are called endoreversible heat engines [46].

At the time of publication of reference [28], the observed efficiency (η=0.36) of a coal-fired power plant was close enough to $\eta_{CA}=0.40$ to make the endoreversible model convincing. However, modern steam power plants typically exceed $\eta_{CA}$ substantially—up to η=0.48. Fu et al. [47] have shown that the entropy production in the heater and the condenser plays a relatively small role in reducing the efficiency of real steam power plants, in accordance with the presented in Section 4.

## 8. Discussion

Two hundred years after their publication, Carnot’s Réflexions represent a genuine foundation of thermodynamics. They deserve more attention, especially in physics education. The theoretical separation of irreversible processes from a real heat engine cycle not only provides an ideal engine that nobody could build but also a quite useful method to improve real engines. Irreversible processes should be reduced wherever possible. This is not only true for heat engines. Reducing useless irreversible processes is really what we mean when we talk about saving energy. The concept of irreversibility is easier to understand for both laymen and students of thermodynamics because it does not rely on the technical term energy.

Entropy is the modern equivalent of Carnot’s term calorique. Together with temperature, thermodynamic problems can be solved qualitatively on the basis of only two physical quantities. In particular, it is possible to optimize heat engines by reducing irreversible processes, i.e., by minimizing entropy production.

The waterfall analogy is based on a substance-like conception of entropy. It is more suitable for teaching than an abstract argument with a process quantity (*Q*) commonly called heat. While the ultimate goal is to understand entropy as a physical quantity rather than a substance, the intermediate step is to understand the need for a second thermal quantity besides temperature. The analogy of a waterfall illustrates the origin of waste heat from an engine. Entropy goes into the engine and must come out at a lower temperature.

Carnot invented the heat pump and the combined cycle gas and steam engine as theoretical concepts. These became mature technologies and are cornerstones of today’s energy economy. He should be famous for each of these, but, in fact, his contributions are almost unknown. To summarize in the words of Coopersmith [24], *Carnot always had an intuition, a hunch, for what was right.* The bicentenary of Carnot’s Réflexions is a good opportunity to delve deeper into the history of thermodynamics.

## Data Availability

The original contributions presented in the study are included in the article, further inquiries can be directed to author.
